# COVID-19 Self-Test Data: Challenges and Opportunities — United States, October 31, 2021–June 11, 2022

**DOI:** 10.15585/mmwr.mm7132a1

**Published:** 2022-08-12

**Authors:** Matthew D. Ritchey, Hannah G. Rosenblum, Kim Del Guercio, Matthew Humbard, Steven Santos, Jason Hall, Jasmine Chaitram, Reynolds M. Salerno

**Affiliations:** ^1^CDC COVID-19 Emergency Response Team; ^2^Division of Health Informatics and Surveillance, Center for Surveillance, Epidemiology, and Laboratory Services, CDC; ^3^Epidemic Intelligence Service, CDC; ^4^Deloitte Consulting LLP, Atlanta, Georgia; ^5^Administration for Strategic Preparedness and Response, U.S. Department of Health and Human Services, Washington, D.C.; ^6^Division of Laboratory Systems, Center for Surveillance, Epidemiology, and Laboratory Services, CDC.

Self-tests* to detect current infection with SARS-CoV-2, the virus that causes COVID-19, are valuable tools that guide individual decision-making and risk reduction^†^ (*1*–*3*). Increased self-test use (*4*) has likely contributed to underascertainment of COVID-19 cases (*5*–*7*), because unlike the requirements to report results of laboratory-based and health care provider–administered point-of-care COVID-19 tests,^§^ public health authorities do not require reporting of self-test results. However, self-test instructions include a recommendation that users report results to their health care provider so that they can receive additional testing and treatment if clinically indicated.^¶^ In addition, multiple manufacturers of COVID-19 self-tests have developed websites or companion mobile applications for users to voluntarily report self-test result data. Federal agencies use the data reported to manufacturers, in combination with manufacturing supply chain information, to better understand self-test availability and use. This report summarizes data voluntarily reported by users of 10.7 million self-tests from four manufacturers during October 31, 2021–June 11, 2022, and compares these self-test data with data received by CDC for 361.9 million laboratory-based and point-of-care tests performed during the same period. Overall trends in reporting volume and percentage of positive results, as well as completeness of reporting demographic variables, were similar across test types. However, the limited amount and quality of data reported from self-tests currently reduces their capacity to augment existing surveillance. Self-tests provide important risk-reduction information to users, and continued development of infrastructure and methods to collect and analyze data from self-tests could improve their use for surveillance during public health emergencies.

CDC analyzed COVID-19 self-test result data voluntarily reported by users of tests produced by four manufacturers** to describe available data and related metrics compared with those from COVID-19 laboratory-based and point-of-care nucleic acid amplification tests (NAATs) and point-of-care antigen tests reported by states and territories through the COVID-19 Electronic Laboratory Reporting (CELR) data system.^††^ Positive NAAT results are considered confirmatory laboratory evidence for SARS-CoV-2 infection, and are the main test type used to track national and local community transmission levels (*8*). Positive point-of-care antigen test results meet the case definition for probable SARS-CoV-2 infection and are used less frequently for national surveillance. Data were analyzed for tests conducted during October 31, 2021–June 11, 2022, to assess the following metrics: 1) weekly testing volume (number of test results reported); 2) 7-day average percentage of positive test results (the number of positive tests reported divided by total tests reported within a 7-day period); and 3) overall completeness of reporting of critical demographic variables (age, sex, and race or ethnicity). CDC does not receive information on patients’ actual name, address, telephone number, or email for test results; however, completeness of self-test obfuscated values (i.e., the fields are coded as having information but the values [e.g., name] are not provided) was able to be assessed based on data obtained during May 25–June 3, 2022.^§§^ This activity was reviewed by CDC and was conducted consistent with applicable federal law and CDC policy.^¶¶^

During October 2021–May 2022, the four manufacturers produced 393.4 million self-tests, representing 15.3% of all self-tests produced for the United States during this period.*** During October 31, 2021–June 11, 2022, users voluntarily reported results of 10,673,837 self-tests through the four manufacturers’ websites or companion mobile applications compared with results of 276,257,710 laboratory-based and point-of-care NAATs and 85,670,213 point-of-care antigen tests reported through the CELR system. For all test types, the peak reported test volume occurred during the week ending January 8, 2022 (Figure 1). During the weeks ending November 6, 2021, and April 23, 2022, the volume of reported laboratory-based and point-of-care NAAT results ranged from 1,947 to 14 times that of self-reported test results, respectively. During the same period, trends in percentages of positive test results were similar across test types; the highest percentage of positive laboratory-based and point-of-care NAAT results (29.1%) and self-tests (17.3%) occurred during the week ending January 8, 2022, and for point-of-care antigen tests (19.8%), occurred during the week ending January 1, 2022 (Figure 2).

**FIGURE 1:**
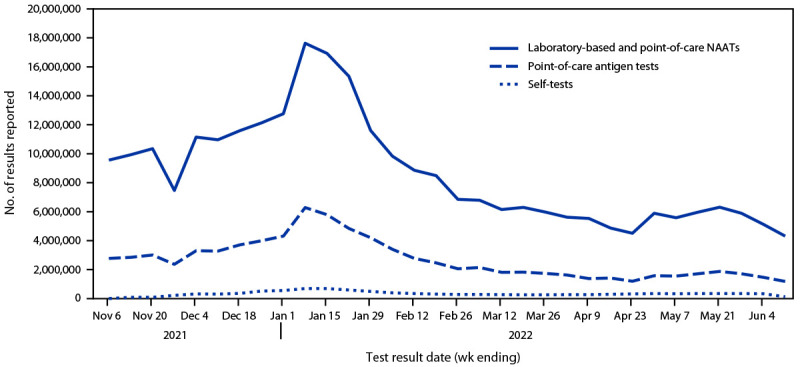
Weekly number of reported results for COVID-19 self-tests,* point-of-care antigen tests, and laboratory-based and point-of-care nucleic acid amplification tests — United States, October 31, 2021–June 11, 2022 **Abbreviation:** NAAT = nucleic acid amplification test. * Self-tests reflect primarily antigen test results but can include NAAT results.

**FIGURE 2:**
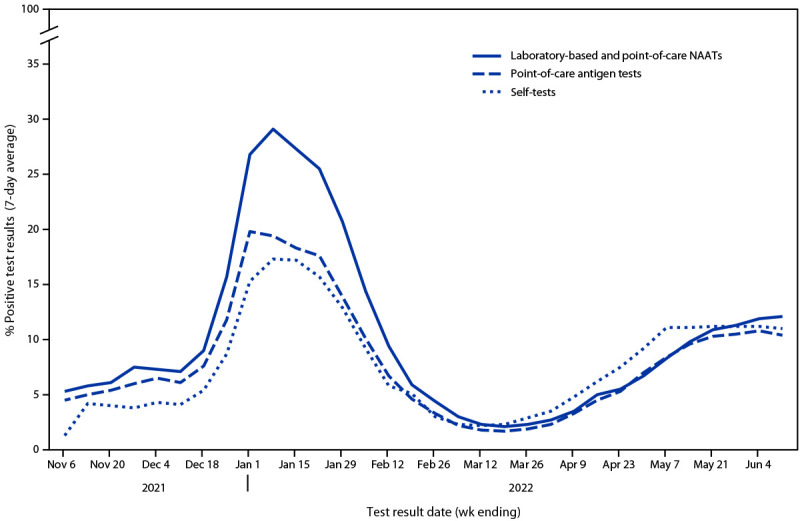
Seven-day average percentage of positive test results reported for COVID-19 self-tests,* point-of-care antigen tests, and laboratory-based and point-of-care nucleic acid amplification tests — United States, October 31, 2021–June 11, 2022 **Abbreviation:** NAAT = nucleic acid amplification test. * Self-tests reflect primarily antigen test results but can include NAAT results.

During October 31, 2021–June 11, 2022, completeness of reporting of demographic information varied across test types and was similar to, but generally higher for laboratory-based and point-of-care tests than for self-tests (Table). For self-test results reported during May 25–June 3, 2022, obfuscated values (i.e., the fields are coded as having information but the values [e.g., name] are not provided) for the customer’s name (first and last) were included in 24.8% of reported self-test results, address was included in 9.8%, telephone number in 17.2%, and email address in 26.6%.

**TABLE:** Completeness of reporting demographic fields for COVID-19 self-test, point-of-care antigen test, and laboratory-based and point-of-care nucleic acid amplification test results — United States, October 31, 2021–June 11, 2022*

Demographic field	% of records with complete information		
	Self-tests^†^	Point-of-care antigen tests	Laboratory-based and point-of-care NAATs
Age	83.1	98.9	97.7
Sex	86.2	92.5	95.4
Race or ethnicity	43.0	58.4	53.2
Name (first and last)*	24.8	NA	NA
Address*	9.8	NA	NA
Telephone no.*	17.2	NA	NA
Email*	26.6	NA	NA

**Abbreviations:** NA = not available; NAAT = nucleic acid amplification test.

* CDC does not receive information on patient’s actual name, address, telephone number, or email for laboratory-based tests, point-of-care tests, or self-tests. Patient contact information is made available on nearly all laboratory-based test and point-of-care test results because the fields are mandated for laboratory reporting; however, these data are only made available to local and state public health agencies to support case investigations and are not included in the data sent to CDC via the COVID-19 Electronic Laboratory Reporting system. Self-test users can include personal identifiable information when they submit results to manufacturers; however, these fields are obfuscated for CDC use (i.e., the field is coded as having information but the value [e.g., name] is not provided). Data for obfuscated patient contact information data elements for self-test results were only available for analysis during May 25, 2022–June 3, 2022.

^†^ Self-tests reflect primarily antigen test results but can include NAAT results.

## Discussion

During October 2021–May 2022, approximately 393 million self-tests were produced by the four manufacturers assessed in this study. Although not all self-tests produced by these manufacturers were distributed, purchased, and used, the 10.7 million results voluntarily reported by users and made available for public health surveillance likely reflect a small fraction of the number of self-tests used. This finding indicates that throughout the COVID-19 pandemic, including during the Omicron variant surge period (December 2021–February 2022) covered by this analysis (*6*,*7*), underascertainment of cases has occurred (*5*). Underascertainment might be attributed to multiple factors, including the lack of formal mechanisms to enable reporting of self-test results to public health authorities and persons with mild or no symptoms not seeking testing or health care.

Self-tests provide another option for persons seeking accessible testing and remain an important tool to guide individual decision-making and risk reduction. Mandating reporting of all self-test results to public health authorities is not practical and could negatively affect acceptability and use of self-tests, which would be detrimental to minimizing disease spread. Although the increase in self-testing (*4*) might result in underascertainment of total case counts, this analysis indicates that the NAAT data captured via CELR, combined with case data, remain robust and continue to track trends in community transmission.^†††^ In addition, persons with more severe disease are probably more likely to receive a NAAT when seeking care in outpatient or inpatient settings, and national surveillance primarily focuses on these cases. Furthermore, other types of surveillance data provide insights into aspects of disease burden such as demands on health care systems, highly or disproportionately affected populations, and severity indicators. Therefore, even without self-testing result data being formally included in national surveillance efforts, the integrated, whole-of-government surveillance activity for the COVID-19 pandemic^§§§^ remains strong, incorporating data from various sources, including case surveillance, laboratory testing, syndromic surveillance, genomics testing, hospitalizations, health care use, supply chain capacities, school data, wastewater surveillance, vital statistics, and vaccination.

Current limitations in self-test data reduce their usefulness to guide public health decision-making. Cases based solely on positive self-test results do not meet national guidance for confirmed or probable cases because self-tests are not administered by Clinical Laboratory Improvement Amendments (CLIA)-certified providers (*8*). The quality of the specimen, execution of the self-test, result produced, and person tested are unverified in most instances; therefore, reported interpretation of results cannot be confirmed. Moreover, in contrast to NAATs, self-test specimens cannot be submitted for culturing and viral isolate characterization to identify or describe the prevalence of variants. Voluntary reporting is often anonymous and lacks information (e.g., telephone number) necessary for action, including deduplication, case investigation, or contact tracing. Finally, because of the similarity in trends for percentage of positive test results and demographic completeness across test types, self-test results are currently unlikely to enhance the ability to understand disease transmission trends.

Despite these limitations, public health experts need to continue evaluating self-test data to understand how they can be incorporated into future surveillance models. Additional analyses can explore several factors: how communities are using and reporting self-tests, equitable access to self-tests, what factors drive decisions to report results, and representativeness of findings; how often positive self-test results lead to isolation, pursuit of treatment, or confirmation of result with laboratory-based testing; and to what degree self-testing is replacing testing in more traditional settings.

Anticipating the potential importance of self-test data for public health and the growing demand to shift testing outside of care and to individual persons, federal agencies have been building relationships with test manufacturers to enable data transmission for public health use. For example, CDC, through partnerships with the U.S. Digital Service, the National Institutes of Health, the Administration for Strategic Preparedness and Response, and the Association of Public Health Laboratories, worked with manufacturers to advise on data to be collected and supported development of data reporting and data transportation capabilities and sharing of self-test data for broad public health use. In addition, the National Institutes of Health, through their RADx Mobile Application Reporting through Standards (MARS) program, is focusing on leveraging data standards to enhance data harmonization, capture, transmission, and reporting for self-tests for clinical and public health use.^¶¶¶^ Furthermore, certain jurisdictions are leveraging anonymous exposure notification systems that use voluntarily reported test result information, including for self-tests, to notify close contacts of potential COVID-19 exposures.

The findings in this report are subject to at least two limitations. First, self-test data were available from only four manufacturers and from users who voluntarily reported results, representing only approximately 3% of the total self-tests produced by these manufacturers and 0.4% produced by all manufacturers during the period; therefore, these data might not be representative of all self-tests used. Second, data completeness was based on presence of any value and not valid values, and personally identifiable information assessment only captured data for a short period; therefore, estimates provided might not represent overall data quality.

Established surveillance based on NAAT testing is in place that can monitor trends in the spread and effects of COVID-19 within communities. However, during the COVID-19 pandemic, self-tests have become an important public health tool to guide individual decision-making. Persons who use self-tests should be encouraged to report results to their health care providers, who can ensure that they receive additional testing, counselling, and medical care, as clinically indicated. Limitations in currently available self-test data limit their value for present public health COVID-19 surveillance. Continued development of infrastructure and methods to collect and analyze self-test data could improve their value for surveillance purposes during future public health emergencies.

**Summary**

**What is already known about this topic?**

COVID-19 self-test use has increased but reporting of results is not required.

**What is added by this report?**

During October 31, 2021–June 11, 2022, 10.7 million test results were voluntarily reported by users of four manufacturers’ self-tests; during that period, 361.9 million laboratory-based and point-of-care test results were reported. Completeness of reporting demographic variables and trends in percent positivity were similar across test types.

**What are the implications for public health practice?**

Self-tests are a valuable risk-reduction tool that can guide individual actions, but they currently offer limited utility in enhancing public health surveillance. Laboratory-based and point-of-care test result data, in combination with other COVID-19 surveillance information, continue to provide strong situational awareness.

## Footnotes

* The first self-test was authorized by the Food and Drug Administration (FDA) for emergency use in December 2020. As of May 2022, FDA had authorized 20 self-tests (https://www.fda.gov/medical-devices/coronavirus-covid-19-and-medical-devices/home-otc-covid-19-diagnostic-tests). Self-tests are also referred to as home tests, at-home tests, or over-the-counter tests. Self-test data reflect primarily antigen test results but can include nucleic acid amplification test (NAAT) results.

†
https://www.cdc.gov/coronavirus/2019-ncov/testing/self-testing.html

§ Laboratory-based and point-of-care NAAT and antigen test results were identified and classified based on Logical Observation Identifiers Names and Codes identifiers. Laboratory-based and point-of-care test data include NAAT results; setting type for NAAT administration cannot be distinguished based on available data. Point-of-care test result data also include antigen tests administered in settings operating under a Clinical Laboratory Improvement Amendments (CLIA) certificate of waiver. Reporting of all NAAT results is required of facilities with CLIA certification to perform moderate- or high-complexity tests; however, reporting of negative results for point-of-care antigen test results is no longer required, which might artificially inflate percent positivity calculations. https://www.cdc.gov/coronavirus/2019-ncov/downloads/lab/HHS-Laboratory-Reporting-Guidance-508.pdf

¶ As part of their Emergency Use Authorization request submission to FDA, self-test manufacturers were requested to describe how all test users could report all test results to public health and other authorities to whom reporting was required, in accordance with local, state, and federal requirements. In addition, some state and local jurisdictions also established mechanisms for persons to voluntarily report self-test results.

** The four manufacturers send individual self-test result data voluntarily reported by customers to the Association of Public Health Laboratories Informatics Messaging Services platform via ReportStream, and deidentified versions of the data are then made available to CDC within HHS Protect. https://reportstream.cdc.gov/; https://public-data-hub-dhhs.hub.arcgis.com/

††
https://www.cdc.gov/elr/index.html

§§ CDC does not receive information on patient’s actual name, address, telephone number or email for laboratory-based tests, point-of-care tests, or self-tests. Patient contact information is made available on nearly all laboratory-based test and point-of-care test results because the fields are mandated for reporting; however, these data are only made available to local and state public health agencies to support case investigations and are not included in the data sent to CDC via the COVID-19 Electronic Laboratory Reporting system. Self-test users can include personal identifiable information when they submit results to manufacturers; however, these fields are obfuscated for CDC use (i.e., the field is coded as having information but the value [e.g., name] is not provided).

¶¶ 45 C.F.R. part 46, 21 C.F.R. part 56; 42 U.S.C. Sect. 241(d); 5 U.S.C. Sect. 552a; 44 U.S.C. Sect. 3501 et seq.

*** Data on self-test production (defined as the number of tests developed and available for U.S. distribution), overall and for the four manufacturers included in this analysis, were provided by the Office of the Assistant Secretary for Preparedness and Response. Combined monthly production totals for the four manufacturers (other manufacturers), in millions were October 2021: 22.3 (29.9); November 2021: 30.9 (81.0); December 2021: 40.6 (230.7); January 2022: 48.4 (356.8); February 2022: 65.5 (920.7); March 2022: 60.3 (358.6); April 2022: 57.4 (106.8); and May 2022: 68.0 (98.9).

†††
https://covid.cdc.gov/covid-data-tracker/#trends_dailycases

§§§ https://covid.cdc.gov/covid-data-tracker; https://data.cdc.gov/browse?tags = covid-19; https://www.healthdata.gov/browse?tags = hhs+covid-19

¶¶¶
https://www.nibib.nih.gov/covid-19/radx-tech-program/mars
